# The role of type I IFN in autoimmune and autoinflammatory diseases with CNS involvement

**DOI:** 10.3389/fneur.2022.1026449

**Published:** 2022-11-10

**Authors:** Sylvia Raftopoulou, Anna Rapti, Dimitris Karathanasis, Maria Eleftheria Evangelopoulos, Clio P. Mavragani

**Affiliations:** ^1^Department of Physiology, Medical School, National and Kapodistrian University of Athens, Athens, Greece; ^2^First Department of Neurology, National and Kapodistrian University of Athens, Aeginition Hospital, Athens, Greece

**Keywords:** type I IFN, central nervous system, multiple sclerosis, neuropsychiatric lupus (NPSLE), type I interferonopathies

## Abstract

Type I interferons (IFNs) are major mediators of innate immunity, with well-known antiviral, antiproliferative, and immunomodulatory properties. A growing body of evidence suggests the involvement of type I IFNs in the pathogenesis of central nervous system (CNS) manifestations in the setting of chronic autoimmune and autoinflammatory disorders, while IFN-β has been for years, a well-established therapeutic modality for multiple sclerosis (MS). In the present review, we summarize the current evidence on the mechanisms of type I IFN production by CNS cellular populations as well as its local effects on the CNS. Additionally, the beneficial effects of IFN-β in the pathophysiology of MS are discussed, along with the contributory role of type I IFNs in the pathogenesis of neuropsychiatric lupus erythematosus and type I interferonopathies.

## Introduction

Interferons (IFNs) are a group of functionally related cytokines of innate immunity with antiviral, antimicrobial, and immunomodulatory activities (1). Three major types of IFNs are recognized: type I, type II, and type III (2). Type I IFNs were first recognized by Isaacs and Lindenmann in 1957 (3) and since then, a growing body of evidence supports a central role for type I IFNs in antiviral immune responses and in the pathogenesis of various autoimmune diseases (4). Type I IFNs are partitioned into several subclasses (IFN-α, β, δ, ω, ε, τ, ζ, λ, and κ) with the subgroup of IFN-α being further divided into 13 subtypes which are encoded by 13 homologous genes, located on chromosome 9 (9p22-9p21) (5). Upon viral infection, almost all cells secrete type I IFNs (typically IFN-α and IFN-β) with IFN-α being secreted primarily by plasmacytoid dendritic cells (pDCs), whereas epithelial cells, phagocytes, DCs, and fibroblasts can secrete IFN-β (1).

Physiologically, membrane-bound or cytoplasmic pattern-recognition receptors (PRRs) are responsible for detecting microbial products such as lipopolysaccharide (LPS) and endogenous or exogenous nucleic acids (6). In pDCs, upon stimulation of the endosomal PRRs such as the toll-like receptors (TLR)-7/8/9 the myeloid differentiation factor 88 (MyD88) is activated, forming a complex with interleukin-1 (IL-1) receptor-associated kinase 1 (IRAK1) and IRAK4 which in turn phosphorylates the interferon regulatory factor 5 (IRF5) and IRF7 acting as transcription factors for IFN-α production (7–9). In other cell populations such as fibroblasts, macrophages, and epithelial cells, cytosolic PRRs such as RIG1-like receptors (retinoic acid-inducible gene 1) and MDA5 (melanoma differentiation association protein 5) sense RNA, while cGAS (cyclic GMP–AMP synthase) is able to detect DNA, leading to the activation of stimulator of interferon genes (STING) and type I IFN-β production (4, 10).

Upon their secretion, type I IFNs bind to the IFN-α/β receptor (IFN-AR), a cell surface receptor consisting of two subunits, IFNAR and IFNAR1 leading to the intracellular autophosphorylation and activation of the Janus kinase 1 (JAK1) and tyrosine kinase 2 (TYK2) and the subsequent phosphorylation of signal transducer and activator of transcription 1 (STAT1) and STAT2. Ultimately, STAT1 and STAT2 form a heterodimer that binds to IRF9 leading to the formation of IFN-stimulated gene factor 3 (ISGF3), which in turn translocates to the nucleus and stimulates the upregulation of a plethora of interferon-stimulated genes (ISGs) (8). ISGs products are involved in controlling pathogens, while they are also found to be upregulated in many systemic autoimmune diseases (11). Given the magnitude of type I IFNs implication in health and disease, we sought to examine in this review, the complex relationship of type I IFNs and the central nervous system (CNS) under physiological conditions and in selected autoimmune and autoinflammatory diseases with CNS involvement such as multiple sclerosis (MS), neuropsychiatric systemic lupus erythematosus (NPSLE), and type I interferonopathies.

### Type I IFN in CNS: An overview

It is of utmost importance that the CNS, similarly to any tissue, is protected against exogenous or endogenous threats such as pathogens and tumors by a well-functioning immune system. Importantly, infection of the CNS is a major challenge since neurons constitute an irreplaceable cell population that should be maintained despite potential insults (12). To minimize neuronal damage as a result of an infection, the communication between peripheral blood and CNS is restricted by the blood–brain barrier (BBB) and the blood–cerebrospinal fluid barrier (BCSFB) to prevent the entry of noxious stimuli in the brain (13, 14).

Type I IFNs have been implicated in various processes within the CNS, including the prevention of viral invasion. Endogenously produced type I IFN has been shown to confer neuroprotection by preventing viral entry in the CNS through IFNAR-mediated regulation of BBB permeability (15, 16). Indeed, mice deficient for IFNAR were more susceptible to CNS infections when exposed to neurotropic viruses, compared to wild-type mice (17). Nevertheless, when CNS infections do occur, they are followed by type I IFN production, as these cytokines are involved in the first-line defense against infection in the periphery but also the CNS (18). These findings stress the highly protective and antiviral role type I IFNs have in the CNS.

Beyond their antiviral properties, the type I IFN/IFNAR axis seems to play a key role CNS homeostasis and normal brain function suggested by the fact that mice deficient in IFNAR present defects in neuronal autophagy, cognitive function, and synaptic plasticity (19–22). In contrast, other reports suggested that induction of type I IFN signaling in the CNS hinders the vascular repair process following traumatic brain injury or cerebrovascular injury which is further associated with BBB leakage and failure to restore cognitive–motor function (23). Moreover, the overexpression of IFN-β in the CNS of adult wild-type mice transforms the microglial transcriptional signature to induce a process similar to aging leading to impaired cognitive performance (24). Along the same lines, neuronal type I IFN expression was shown to be modulated by alpha-synuclein, a protein expressed predominantly in neurons, in association with neurodegenerative diseases such as Parkinson's disease (25). Taken together, these findings highlight the complex role of type I IFN in CNS pathophysiology.

### CNS cellular populations involved in type I IFN production

**Figure 1:**
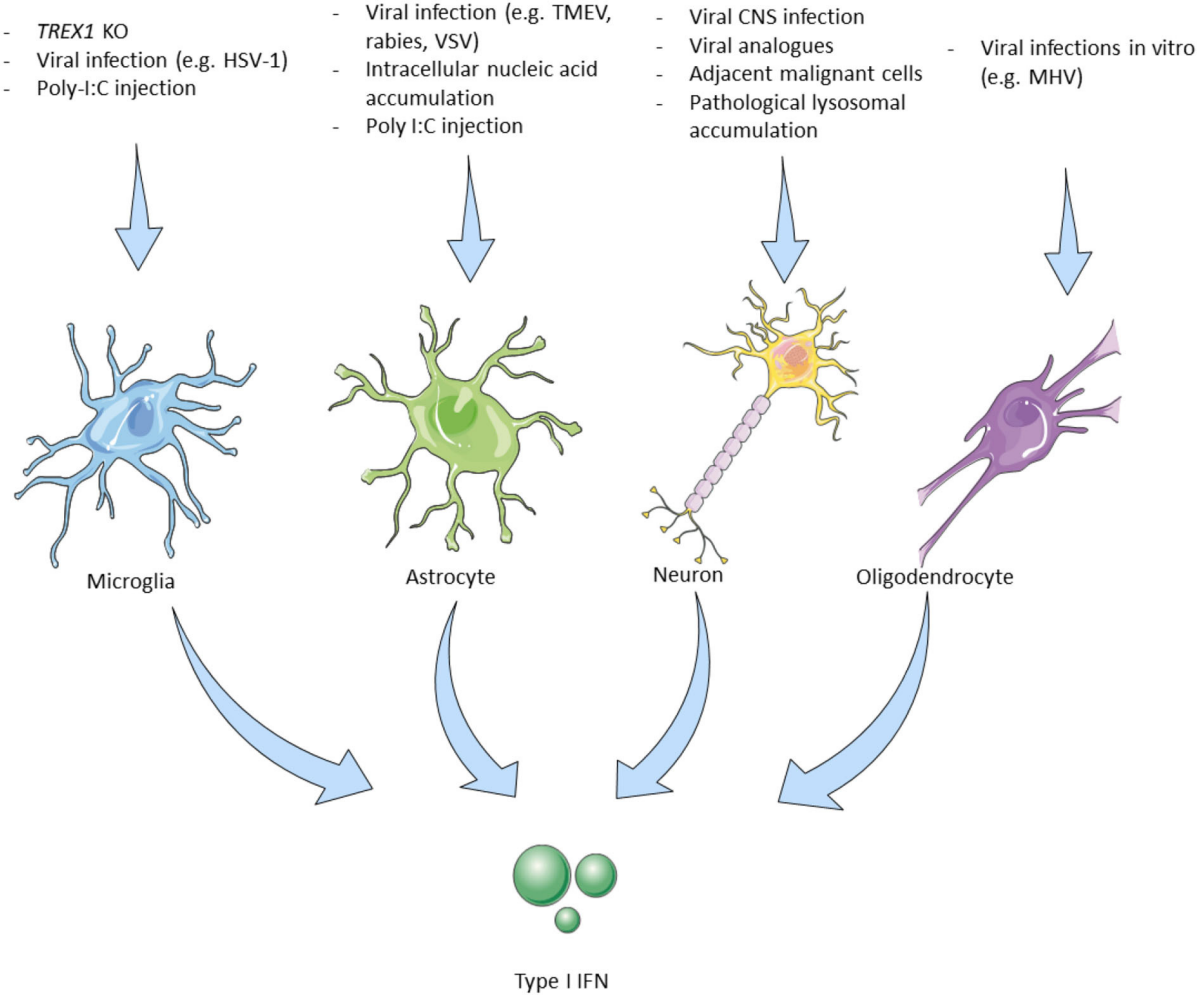
Triggers leading to type I interferon production by cells of the central nervous system. Microglia, The main type I IFN producer cells in the CNS. Trex1 homozygous deletion selectively in murine microglial cells induced type I IFN production. Intrathecal poly I:C induces IFN-β production in EAE with subsequent therapeutic effects (28). Microglial cells are the main producers of cGAS–STING-dependent type I IFN in animal models of HSV-1 encephalitis (29). Astrocytes, Murine-derived astrocytes can be experimentally triggered by viral and microbial infection analogs to produce type I IFN. IFN-β mainly produced by astrocytes in different CNS viral infections in mouse models (30). Neurons, *In vitro* murine and human neurons produce IFN-β upon stimulation with viruses or their analogs (31). Viral encephalitis also causes neuronal type I IFN production but only by 3% of the infected murine neurons *in vivo*. Some neurons adjacent to GBM cells express the IFN-β dependent PD-L1 which improves survival (32). IFN-β -/- neurons have a worse prognosis. Specific cortical neurons in patients with Gaucher disease affecting the CNS show elevated IFN-β production (33). Oligodendrocytes, MHV infection *in vitro* (mouse-derived cells) induces type I IFN production. MHV infection (JHM strain) in live mice did not lead to IFN-I production (18). IFN, interferon; CNS, central nervous system; Trex1, three prime repair exonuclease 1; EAE, experimental autoimmune encephalomyelitis; HSV, herpes simplex virus; cGAS, cyclic GMP–AMP synthase; STING, stimulator of type I IFN genes; TLR, toll-like receptor; AGS, Aicardi Goutières syndrome; LPS, lipopolysaccharides; TMEV, Theiler's murine encephalomyelitis virus; MHV, mouse hepatitis virus; GBM, glioblastoma multiforme; PD-L, program death-ligand; GBA1, glucosylceramidase β 1.

Plasmacytoid dendritic cells are the professional type I IFN-producing cells in the periphery, as they produce large amounts of IFN-α upon their activation by dedicating 60% of new transcriptional activity to make type I IFNs (26). However, it is reported that under physiological conditions, pDCs are absent from the brain, due to the CNS being an immune-privileged site (27). Even though the CNS lacks access to professional producers of type I IFN, these cytokines are still detected in the CNS, suggesting the ability of CNS cell populations to locally produce and respond to type I IFNs. In the following sections, we summarize the potential of CNS cells to locally produce type I IFNs under physiological conditions or upon stimulation (summarized in Figure 1).

### Microglia

Microglia are the resident immune cells of the brain that undergo rapid change states in response to their environment (34) and are one of the main type I IFN-producing cells in the CNS. In response to nucleic acid accumulation, either due to a 3' prime exonuclease 1 (Trex1) gene deletion—which physiologically degrades dsDNA, ssDNA, and ssRNA (28)—or due to infection with the neurotropic herpes simplex virus 1 (HSV-1) in microglial cells (29), the cGAS-STING pathway is activated, resulting in type I IFN production. Kocur *et al*. studying the role of microglia in experimental autoimmune encephalomyelitis (EAE), the murine model of MS, demonstrated that a subpopulation of activated microglial cells produces the largest amount of IFN-β in EAE (35). Another experimental proof for the role of microglial cells as type I IFN-producing cells came by Khorooshi *et al*., they demonstrated that intrathecal injection of polyinosinic–polycytidylic acid (poly I:C) (a double-stranded RNA analog and TLR3 agonist and therefore a potent inducer of IFN-β), in mice with EAE, led to increased IFN-β expression by parenchymal microglial cells and also had a disease remission effect (36) (Figure 1—Microglia).

### Astrocytes

Astrocytes are specialized glial cells that have a regulatory role within the brain and are implicated in processes such as neurogenesis and synaptogenesis, while they are also responsible for maintaining BBB permeability and controlling extracellular homeostasis (37). They express TLR3 and are also important producers of type I IFN in the CNS (18, 30). Murine astrocytes produce type I IFN upon stimulation with polyribonucleotide, cycloheximide, actinomycin D, and poly I:C (38, 39). Studying different neurotropic viruses in mice including rabies virus, Theiler's murine encephalomyelitis virus, and vesicular stomatitis virus, Pfefferkorn and colleagues showed that although these viruses mainly infect and reproduce in neurons, astrocytes are the main producers of IFN-β in response to these infections. They also suggested that transiently infected astrocytes produce the majority of IFN-β and that this production is mediated by the TLR and RIG-I-like receptors (RLR) signaling cascade (40). In mice infected with La Cross virus, astrocytes were also the major IFN-β producing cells (41) (Figure 1—Astrocytes).

### Oligodendrocytes

Oligodendrocytes are primarily responsible for the maintenance and generation of the myelin sheath that surrounds axons. The precursors of oligodendrocytes have to undergo a well-orchestrated process of proliferation, migration, and differentiation to produce the myelin sheath of axons (42). They reportedly express TLR2 and TLR3, while their ability to produce type I IFN is rather conflicting (18, 43). Li et al. showed that MHV-infected oligodendrocytes express IFN-α/β *via* RIG-I activation *in vitro* (44). On the contrary, overexpression of MDA5, RIG-1, and TLR3 following MHV infection in the JHM strain *in vivo* did not result in type I IFN production, while poly I:C stimulation was not able to induce type I IFN production (43) (Figure 1—Oligodendrocytes).

### Neurons

Neurons, the fundamental units of the nervous system, have also been implicated in the production of type I IFN upon stimulation with various triggers, while they express TLR3, similarly to microglial cells and oligodendrocytes (18, 30). *In vitro* studies demonstrated that murine and human neurons stimulated with poly I:C or viruses (Sendai, rabies viruses), produced IFN-β, (45, 46). In experimental mice, CNS infection with several viruses (Theiler's virus, La Crosse virus encephalitis) led to type I IFN production (31), while rabies virus, although capable of diminishing the type I IFN response by inhibiting the IRF3 pathway, still triggers the small amount of type I IFN production by infected neurons (47). Although IFN-β is shown to downregulate both IFNγ-producing Th1 cells and IL-17-producing Th17 cells, a paradoxical disease exacerbation was witnessed after IFN-β administration in a Th17-induced EAE murine model (48, 49).

Of interest, a study in patients with glioblastoma multiform (GBM) showed that in some cases neurons adjacent to the tumor express the IFN-β induced programmed death-ligand (PD-L)-1, which in turn induces caspase-dependent apoptosis of malignant cells in association with better survival. In accordance with these findings, the deletion of IFN-β in neurons of mice with experimental glioblastoma also led to worst outcomes (32). Finally, in a mouse model of Gaucher disease, a genetic lysosomal storage disease caused by acid-β-glucosidase (glucocerebrosidase) deficiency, it was shown that specific cortical neurons (along with microglia) demonstrated increased IFN-β production (33) (Figure 1—Neurons).

## Type I IFN in the CNS: Association with clinical phenotypes

### Multiple sclerosis

Multiple sclerosis is the prototype CNS autoimmune demyelinating disease. Its pathophysiology has been extensively studied but many pathways remain to be elucidated. Although traditionally considered a predominantly T-cell-mediated inflammation, recent advances, including the high effectiveness of B-cell depletion therapies, have unraveled the importance of humoral immunity as well (50). Innate immunity has recently attracted research interest, especially in progressive forms of the disease with potential therapies expected in the future (51, 52). Regarding type I IFN, it has long been implicated in the pathophysiology of MS mainly because of its protective role, with IFN-β being the oldest approved treatment for relapsing-remitting MS (RRMS) (53). There are currently four IFN-β approved drugs for the treatment of relapsing forms of MS, three of which are administered subcutaneously (SC), IFNβ-1b, IFN-β-1a, and most recently peginterferon β-1a, one intramuscularly: IFN-β-1a (54). Treatment with IFN-β confers a decrease of approximately 30% in the annualized relapse rate (ARR) (55). On the contrary, administration of IFN-γ to patients with MS exacerbates the disease and results in increased numbers of relapses (56). Major advancements in the understanding of MS, and the efficacy of IFN-β as a treatment for the disease, came from studying the EAE model (57). Importantly, EAE has greatly facilitated the understanding of the role that type I IFN plays in the CNS under the MS setting. Although the exact mechanisms through which IFN-β exerts its beneficial role have not been fully elucidated, there are some experimental data pointing to this direction.

**Figure 2:**
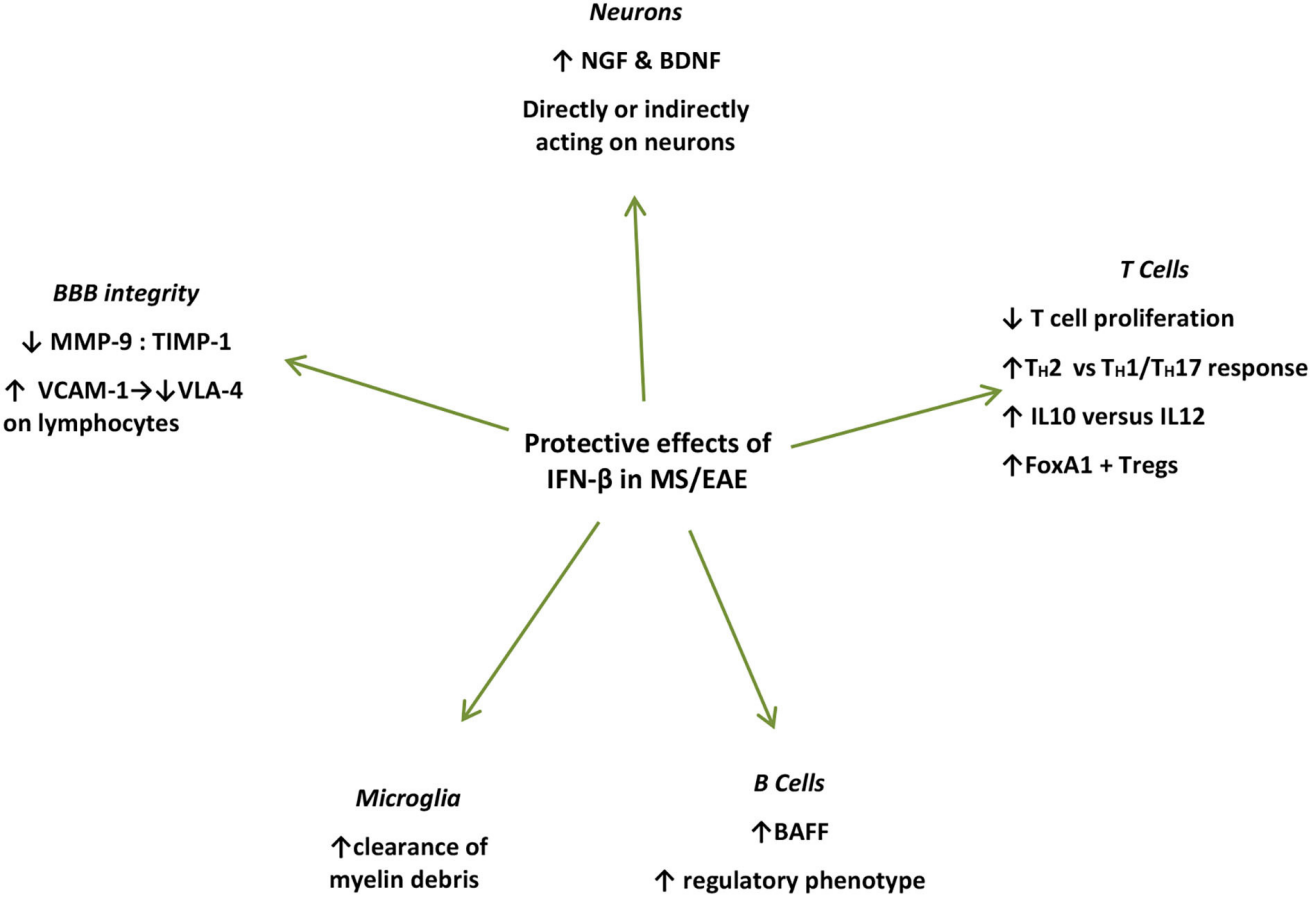
Summary of protective mechanisms of action of endogenously produced or exogenously administered IFN-β during the effector phase of EAE/MS. IFN-β treatment induces NGF in astrocyte cultures (51), and in endothelial cells interacting with IFN-β-treated lymphocytes *in vivo* and *in vitro* (61) IFN-β further induces BDNF production (62). Microglial cells in close proximity to myelin debris produce IFN-β in EAE mice and *in vitro* (35). IFN-β treatment in MS patients induces BAFF and drives B cells toward a regulatory phenotype (63). IFN-β inhibits T-cell proliferation (64), and recombinant IFN-β administered to RRMS patients is associated with increased proliferation of Foxp3+ Tregs as well as FoxA1+ Treg cells (65–67). IFN-β favors Th2 over Th1 responses (66). IFN-β treatment in MS patients leads to increased levels of soluble VCAM1 in their serum leading to a decrease in VLA-4 expression on lymphocytes, interfering with their capacity to bind endothelial cells thereby preventing their diapedeses through the BBB (68). IFN-β further prevents the erosion of the BBB by reducing MMP-9 or by increasing levels (69–71). IFN, interferon; CNS, central nervous system; NGF, nerve growth factor; BDNF, brain-derived neurotrophic factor; BAFF, B-cell activation factor; Treg, regulatory T cells; VCAM-1, vascular cell adhesion molecule-1; VLA-4, very late antigen 4; MMP-9, matrix metalloprotease 9; TIMP-1, TIMP metalloprotease inhibitor 1.

For example, it was shown that IFN-β production within the CNS coincides with the peak of EAE (36) and seems to positively affect the clinical course of the disease, as also implied by the detrimental clinical effects of IFNAR gene ablation in EAE (58, 59). The beneficial IFN-β effect on the CNS was further highlighted as IFN-β producing microglia mediate the clearance of myelin debris *via* phagocytosis (36). This IFN-β-mediated elimination of damaged myelin sheath residues leads to a late-stage suppression of proinflammatory mediators, possibly contributing to favorable results in terms of neuroinflammation control (60) (Figure 2).

Furthermore, with respect to neurons, IFN-β potently induces nerve growth factor (NGF) production in IFN-β-treated astrocyte cultures (72), and in endothelial cells interacting with IFN-β-treated lymphocytes *in vivo* and *in vitro* (61). IFN-β further induces brain-derived neurotrophic factor (BDNF) production in PBMCs from patients with RRMS after 6 months of IFN-β treatment, who show clinical remission for at least 1 year of follow-up (62). However, in these settings, whether these factors act directly on neuronal cells to promote repair or proliferation, remains to be elucidated (61). Moreover, IFN-β was shown to directly promote human neural progenitor cells (NPCs) proliferation and differentiation *in vitro*, in a dose-dependent manner (73) (Figure 2).

IFN-β treatment in patients with MS also induces B-cell activation factor (BAFF) and drives B cells toward a regulatory phenotype (63). BAFF blockade with atacicept, in the setting of RRMS led to disease exacerbation, which further proves the unexpected protective effect of IFN-β-induced BAFF induction (74). Surprisingly, the previously identified risk allele for both MS and SLE of TNFSF13B (GCTGT>A) is linked to increased numbers of circulating B cells and drastically increased levels of soluble BAFF, as well as increased antibody production (75). Furthermore, IFN-β has been shown to inhibit T-cell proliferation (64), while it induces immune cell apoptosis (76). Recombinant IFN-β administered to patients with RRMS is associated with increased proliferation of CD56 ^bright^ natural killer (NK) cells, Foxp3+ regulatory T cells (Treg), as well as FoxA1+ Treg cells, favoring Th2 over Th1 responses (60, 61, 72) (Figure 2).

In addition, IFN-β treatment in patients with MS leads to increased levels of soluble vascular cell adhesion molecule-1 (VCAM-1), which subsequently leads to a significant decrease in VLA-4 expression on lymphocytes, interfering with their capacity to bind endothelial cells thereby preventing their diapedeses through the BBB (68). Finally, IFN-β further prevents the erosion of the BBB by reducing matrix metalloproteinase 9 (MMP-9) levels (69) or by increasing counter-acting tissue inhibitor of matrix metalloproteinase (TIMP-1) levels (63, 74) (Figure 2).

While the aforementioned evidence supports the therapeutic role of type I IFN in the context of neuroinflammation, conflicting data have also emerged to shine the light on some less favorable aspects of this cytokine effect on CNS. For instance, pDCs, the professional type I IFN-producers in the periphery, promote the priming of encephalitogenic T cells during the induction phase of EAE, partially implicating IFN-α/β (77). Despite MS/EAE being traditionally perceived as mainly IFNγ-mediated, deletion of a negative regulator of IFN-α /β, c, in myeloid cells of EAE mice, resulted in uncontrolled type I IFN signaling and white matter microglia activation due to prolonged STAT1 phosphorylation, leading to disease exacerbation (78). Importantly, IFN-I-induced STAT1 is key for priming cellular responses to type II IFN signals (79). Communication between type I and II IFN pathways is constant and their disequilibrium in terms of excessiveness or timing could be rendered as a key contributor to CNS autoimmunity in the context of EAE/MS (80). Interestingly, our group investigated the interferon signature in patients originally presented with MS-like CNS manifestation. A subset of these patients was shown to meet classification criteria for a systemic autoimmune disease (e.g., Sjogren's syndrome, SLE). These patients displayed higher expression of type I IFN-stimulated genes in peripheral blood, compared to the patients who received a final diagnosis of MS or cerebral small vessel disease and so this distinct group may be of potential therapeutic benefit from type I IFN blockade (81).

### Neuropsychiatric lupus erythematosus

SLE is a heterogeneous autoimmune disease with multisystemic presentations and a wide range of clinical and serological manifestations (82). NPSLE is a term used to collectively describe the symptoms of neurological and psychiatric nature present in 80% of patients with SLE (83). These symptoms affect the CNS and peripheral nervous system (PNS) and can range from localized or isolated to diffuse manifestations, while they can also vary in severity. The diagnosis of NPSLE can be challenging due to the lack of disease-specific clinical and laboratory criteria (84). Moreover, the pathogenesis of NPSLE has not been well characterized so far, mainly due to the heterogenicity in the clinical characteristics of patients with NPSLE. However, it is believed that BBB disruption may play a key role here, similarly to MS, whereby immune cells and autoantibodies eventually enter the CNS, possibly promoting neuroinflammation (85). On a genetic basis, a single-nucleotide polymorphism (rs11797) on the *TREX1* gene has been associated with NPSLE as it discriminates patients with NPSLE from patients with SLE without CNS involvement (86).

It is reported that 50–75% of adult patients with SLE have elevated production of type I IFN, giving rise to an increased IFN signature (87), while patients with active SLE benefit from the treatment with anifrolumab, a human monoclonal antibody against type I IFN receptor subunit 1, blocking the activation of type I IFNs (88). However, NPSLE was an exclusion criterion for the clinical trials of anifrolumab (89), possibly due to the lack of evidence that associates NPSLE and the IFN signature. However, there are several studies and animal models of NPSLE that relate the IFN signature to NPSLE.

Among the first groups to hypothesize that type I IFN plays a role in NPSLE pathology was that of Shiozawa in 1992 who demonstrated that IFN-α in CSF of patients with NPSLE was higher compared to those with SLE alone. Moreover, IFN-α levels in the CSF from patients with NPSLE were found to be higher compared to serum IFN-α, suggesting that type I IFN synthesized in the brain may be implicated in the neuropsychiatric manifestations of SLE. Moreover, upon brain autopsy in one of the patients with NPSLE, neuron localization of IFN-α protein and mRNA was detected (90). In addition, Santer *et al*. used a bioassay using pDCs to demonstrate that NPSLE CSF induced significantly higher IFN-α compared with CSF from patients with MS or other autoimmune disease controls (91). Recently, IFN-α was shown to have a moderate correlation (ρ = 0.33; *p* = 0.05) with NPSLE at the onset of neuropsychiatric manifestations (92). However, the exact implication of type I IFN in NPSLE pathophysiology remains to be elucidated.

Numerous animal models have been developed in an effort to study NPSLE and characterize its pathology; however, none is a true depiction of the disease due to its heterogeneous nature [reviewed in (93)]. The Pristane-induced lupus (PIL) model more closely resembles the human NPSLE manifestations. Upon pristane injection, mice develop lipogranulomas containing mononuclear cells and DCs allowing the initiation of the inflammatory response. The model is characterized by a strong IFN signature, autoantibody production, glomerulonephritis, arthritis, and anemia. Among the neuropsychiatric manifestations in this model are learning and memory deficits as well as decreased locomotion (80, 81). The spontaneous NZB/NZW F1 mouse NPSLE model is characterized by splenomegaly, glomerulonephritis, haemolytic anemia, and lymphadenopathy, while learning and memory deficits are among its neuropsychiatric manifestations. Similarly to other models, NZB/NZW F1 mice are characterized by elevated production of IL-6, IFN-γ, and TNF-α, as well as antinuclear antibodies and anti-dsDNA, while their IFN signature is weak (94). Finally, the spontaneous MRL/*lpr* model shares some similarities with the aforementioned models with the neuropsychiatric manifestations such as depression, anhedonia, apathy, and anxiety being more pronounced here (94). Of interest, the intraperitoneal injection of captopril, an angiotensin-converting enzyme (ACE) inhibitor, in MRL*/lpr* mice reduced the ISGs expression in the brain and the periphery as well as IFN-α levels in the plasma. Of interest, microglial activation in the brain was also dampened. After short-term oral captopril treatment, there was a decrease in the depressive-like behavior responses in MRL/*lpr* mice, highlighting the potential therapeutic benefits of ACE inhibitors in treating NPSLE (95).

### Type I interferonopathies with CNS involvement

Autoinflammatory diseases result from immune system dysfunction and mainly affect the innate immune system. This differentiates them from autoimmune diseases such as MS and NPSLE, which are traditionally attributed to a malfunctioning adaptive immune system. Clinically, most autoinflammatory diseases are characterized by symptoms of systemic inflammation. Most significantly, type I interferonopathies that fall under the umbrella of autoinflammatory diseases are characterized by the significant involvement of the CNS, in relation to type I IFN as it will be discussed later.

A strong link between autoinflammatory diseases and the CNS can be seen in type I interferonopathies. The prototypic mendelian disease that demonstrated the potential harm of interferon in humans was firstly described by J. Aicardi and F. Goutières in 1984. They reported a progressive familial encephalopathy in infancy (96), characterized by dysregulated type I IFN pathway and accompanied by IFN-α-producing astrocytes that have neurotoxic effects in the CNS, as subsequently highlighted by the Campbell group (97). Type I interferonopathies are triggered by host nucleic acids, due to the failure of the innate immune system to distinguish self from non-self. Technological advancements have enabled the understanding of type I interferonopathies at least on a genetic basis, and it is now suggested that 38 Mendelian genotypes can be classified as type I interferonopathies (98). The genetic background of interferonopathies with neuroinflammatory manifestations has been greatly investigated. However, the way in which type I interferonopathies present neurological manifestations and whether type I IFNs are directly implicated in this process is greatly understudied. Apart from AGS, for the interferonopathies that were most recently described, there is very little evidence around the origin of type I IFN induction.

Collectively, the dysregulation of type I IFN that eventually leads to interferonopathies may be a consequence of variable mechanisms that are summarized here and are mainly found in the periphery. As seen in AGS, monogenic SLE, trichohepatoenteric syndrome, and X-linked reticulate pigmentary disorder, the accumulation of endogenous nucleic acids in the cytosol that trigger aberrant type I IFN production, result from loss-of-function mutations in genes encoding for enzymes targeting nucleic acid degradation. Furthermore, AGS and monogenic SLE are characterized by alterations in the intracytosolic nucleic acid sensor, which in turn leads to a lowered threshold for IFN production, a mechanism also observed in Singleton–Merten syndrome. Other interferonopathies such as STING-associated vasculopathy with onset in infancy are triggered by a gain of function mutation of positive IFN signaling regulators, leading to continuous activation of the IFN pathway (99).

Diagnostic testing for type I interferonopathies is a novel concept that has not yet reached standard clinical practice. However, the assessment of type I IFN signature has been shown to define a spectrum of inflammatory diseases, related to aberrant type I IFN signaling (100). In addition, IFN-α can be measured through digital enzyme-linked immunosorbent assay technology (98).

Generally, type I IFN is involved in microglial function by regulating the balance between physiological clearance of debris and aberrant phagocytosis. A dysregulation of this balance can lead to neurodegeneration (101). Fetal nervous tissues appear to be more prone to IFN insult, particularly during developmental processes such as neurogenesis and myelination (102). As a consequence, neurological involvement, such as psychomotor retardation and intracerebral calcifications, seen in some interferonopathies such as AGS are reported early on during the disease onset (100). Interestingly, a group of organisms causing congenital fetal infections (toxoplasma, rubella, CMV, and HSV), collectively termed TORCH, also cause intracranial calcifications in neonatal periods, reminiscent of the ones found in interferonopathies (103). We will discuss here the genetic background and immunopathology of interferonopathies with neurological involvement (summarized in Table 1).

**Table 1:** Genetic background and immunopathology of interferonopathies with neuroinflammatory manifestations.

**Interferonopathy**	**Associated genes**	**Immunopathology**	**Clinical manifestations**
Aicardi–Goutières syndrome	*TREX1 RNASEH2B RNASEH2C RNASEH2A SAMHD1 ADAR IFIH1 LSM11 RNU7-1 USP18 STAT2*	Dysregulated nucleic acid sensing resulting in immune activation and aberrant type I IFN production.	Encephalopathy, intracranial calcifications, cerebral atrophy (104).
ISG15 deficiency	*ISG15*	Deficiency in *ISG15* leads to upregulated type I IFN and downregulated type II IFN signaling.	Cerebral calcifications, susceptibility to mycobacterial infection (105).
DNase II deficiency	*DNASE2*	Lack of DNase endonuclease activity results in aberrant sensing of self-DNA resulting in aberrant type I IFN induction.	Anemia, glomerulonephritis, liver fibrosis, cerebral calcifications, white matter (106).
Spondyloenchondrodysplasia (SPENCD)	*ACP5*	Deficiency in ACP5 results in upregulated type I IFN response	Bone lesions, autoimmune thrombocytopenia, SLE, vasculitis, cerebral calcifications, developmental delay (107)
Chronic atypical neutrophilic dermatosis with lipodystrophy and elevated temperature (CANDLE).	*PSMB8*	Immuno-proteosomal dysfunction possibly leads to the accumulation of misfolded or damaged proteins resulting in type I IFN induction.	Recurrent fever, rashes, arthralgia, progressive lipodystrophy, cerebral calcifications (108)

### Aicardi–goutières syndrome

AGS is characterized by basal ganglia calcifications, CSF lymphocytosis, as well as increased levels of type I IFN in the CSF (109). There are seven AGS genetic subgroups (AGS1-7), based on the mutations found in the following genes: 3′-5′ DNA exonuclease-*TREX1, RNASEH2B, RNASEH2C*, and *RNASEH2A*, SAM and HD domain-containing deoxynucleoside triphosphate triphosphohydrolase *1-SAMHD1*, and adenosine deaminase acting on RNA *1-ADAR1*, as well as on the RNA sensor IFN-induced helicase C domain-containing protein *1-IFIH1* (101, 110). Mutations in *TREX1* and the genes encoding the RNASEH2 complex led to the hypothesis that these proteins are involved in nucleic acid ‘debris’ clearance and their dysfunction could result in the failure of such clearance thereby leading to an innate immune response that would physiologically be induced by viral nucleic acids (93, 111). Deficiency in *TREX1* results in the accumulation of intracellular ssDNA. As demonstrated by Stetson and colleagues, *Trex1*-null mice experience the activation of the TLR-independent cytosolic pathway by ssDNA, resulting in type I IFN production (112). However, the question still remains regarding the source of IFN in the CNS.

As mentioned, astrocytes and microglia are key producers of IFN-α within the CNS when responding to viral infection or synthetic poly I:C treatment. Post-mortem studies of patients with AGS revealed co-localization of the GFAP astrocyte marker and IFN-α along with the cytokine CXCL10 (113). What are more, investigations led by Campbell's group utilizing a transgenic mouse model expressing IFN-α in astrocytes specifically (GFAP-IFN), resulted in mice developing a clinical phenotype that overlapped with AGS individuals but lacked the genetic basis of AGS. Importantly, these transgenic mice developed encephalopathy, seizures, and calcium deposits in the basal ganglia, clinical characteristics matching AGS (97). In favor of this theory is the *in vitro* model of astrocytes exposed to poly I:C that exhibits an innate immune response with elevated cytokines such as IFN-α (114). Specifically, when treated with IFN-α for 3 weeks, these astrocytes showed reduced proliferation along with downregulation of genes and proteins crucial for white matter maintenance. In addition, withdrawal from IFN-α for 7 days did not rescue or revert the aforementioned effect (114). This evidence can collectively support the idea of IFN-α-producing astrocytes as a probable mediator in the pathogenesis of AGS.

In terms of clinical characteristics and in the context of early-onset AGS, delay in psychomotor development and liver anomalies are present since birth whereas in the context of later-onset AGS, after a small window of normal development, cognitive and developmental delay become evident (115). Neuroimaging greatly facilitates the diagnosis of AGS, in which brain atrophy, white matter hyperintensities (WMH), and intracranial calcifications are present (116). Extra-neurological symptoms are also prominent in patients with AGS with the skin being most commonly (35%) involved in the form of chilblain-like lesions localized in fingers and toes. In addition, thrombocytopenia, hepatosplenomegaly, psoriasis, and interstitial lung disease (ILD) can also manifest in the context of AGS (101). Taking into account that patients with AGS have increased IFN-α in CSF and peripheral blood, Rice and colleagues proposed in 2013 that the interferon signature could facilitate the diagnosis of AGS, as a possible biomarker. Indeed, the upregulation in expressions of *IFI27, IFI44, IFIT1, ISG15, RSAD2, and SIGLEC1* was confirmed in the peripheral blood of patients with AGS (98, 99).

### ISG15 deficiency

*ISG15* deficiency is a moderately severe interferonopathy. ISG15 is an intracellular IFN-α/β inducible ubiquitin-like modifier which covalently binds other proteins, a process called ISGylation. Through a loss-of-function mutation, ISG deficiency in humans leads to increased type I IFN signaling and decreased type II IFN signaling. Zhang and colleagues demonstrated that patients with *ISG15*-deficient displayed clinical signs of enhanced IFN-α/β immunity as seen in AGS and spondyloenchondrodysplasia (SPENCD) such as cerebral calcifications (nearly 100% of patients with ISG15) and sporadic reports of seizures. It was further shown by the same group that the absence of intracellular ISG15 prevents the accumulation of USP18 thereby sustaining the effect of IFN-α/β inflammation (105, 117). ISG15 also acts as an extracellular inducer of type II IFN and, therefore, its deficiency is subsequently associated with increased susceptibility to mycobacterial disease (117). It was later shown that myeloid cells (monocytes and DCs) were the primary responders to IFN-I-mediated inflammation in the peripheral blood of patients with ISG15 deficiency (118). However, the role of CNS cells in mediating the type I IFN inflammation in the context of ISG deficiency remains to be elucidated.

#### DNase II deficiency

Deoxyribonuclease (DNase) II is a lysosomal endonuclease that facilitates the degradation of extracellular DNA debris generated by homeostatic erythropoiesis and apoptosis (119). DNase II deficient mice accumulate a lethal amount of undigested DNA in the lysosomes of macrophages which chronically induces type I IFN production resulting in lethal perinatal anemia (106). DNase II deficiency is a distinct clinical entity of autoinflammation and elevated type I IFN signaling. Neurological manifestations include once more cerebral calcifications and WMH (117).

#### Spondyloenchondrodysplasia

Spondyloenchondrodysplasia (SPENCD) is a rare autosomal recessive skeletal dysplasia that manifests itself through vertebral dysplasia and enchondroma-like radiolucent metaphyseal lesions of the long bones, while affected individuals are presented with short-trunked short stature (107). Among other clinical manifestations, neurological involvement such as intracranial calcification and immune dysregulation manifesting through autoimmune diseases or immunodeficiency are also present (120). Loss-of-function mutations in the *ACP5* gene (117) which encodes tartrate-resistant acid phosphatase (TRAP) was identified as the genetic cause of SPENCD. As a consequence, the deficit in TRAP leads to elevated levels of phosphorylated osteopontin, which in turn results in dysregulated endochondral ossification but also increased type I IFN signature (120). However, very little is known regarding the induction of type I IFN signature in SPENCD and specifically the way it affects the CNS.

#### Chronic atypical neutrophilic dermatosis with lipodystrophy and elevated temperature

Chronic atypical neutrophilic dermatosis with lipodystrophy and elevated temperature (CANDLE) syndrome is an autoinflammatory disorder characterized by recurrent fevers, purpuric annular plaques, acral pernio-like lesions, periorbital violaceous oedema, lipodystrophy, arthralgias, anemia, and elevated inflammatory markers (121). Similarly to other interferonopathies, cerebral calcifications and rarely aseptic meningitis have also been reported. In addition, the elevated type I IFN signature found in patients with CANDLE may be a key mediator of the inflammatory response and serve as a therapeutic target (108). CANDLE is now classified under the subcategory of interferonopathies called proteasome-related autoinflammation (PRAAS1) and is called PRAAS1 (117). The ubiquitin-proteasome system (UPS) is involved in various cellular functions such as misfolded protein clearance, MHC class I antigen processing, and others (122). Particularly, the loss-of-function mutation in proteasome 20S subunit beta 8 (PSMB8) (117), associated with PRAAS1, results in autoinflammation dominated by a prominent type I IFN gene signature due to impaired proteasome activity and perturbing protein homeostasis. However, the exact mechanism is not fully understood (108).

## Discussion

Since their discovery in 1957, interferons have been implicated in numerous pathophysiological conditions while they have also paved the way for many therapeutic interventions. Here, we summarized the implication of type I IFN in autoimmune and autoinflammatory diseases affecting the CNS. In the past decade, substantial evidence has aided our understanding of the way type I IFN signaling may protect the CNS against viral infection. More importantly, great progress has been made in elucidating how dysregulations in this pathway can lead to neurological diseases. A plethora of observations supports the protective effect of type I IFN in the context of MS and its murine disease analog EAE. On the other hand, NPSLE and type I interferonopathies highlight the detrimental effects of type I IFN on several organ systems, including the CNS, for patients affected by these diseases. Taking everything into account, we conclude that a disproportionate enhancement in type I IFN signaling can lead to autoinflammatory CNS manifestations, whereas peripherally administered type I IFN-β can be beneficial at least for a subset of patients with MS.

## Author contributions

CM and ME conceived, designed, and supervised the review. SR, AR, and DK drafted the manuscript. CM and SR edited the manuscript. All authors critically reviewed the manuscript and agreed to its published version.

## Conflict of interest

The authors declare that the research was conducted in the absence of any commercial or financial relationships that could be construed as a potential conflict of interest.

## Publisher's note

All claims expressed in this article are solely those of the authors and do not necessarily represent those of their affiliated organizations, or those of the publisher, the editors and the reviewers. Any product that may be evaluated in this article, or claim that may be made by its manufacturer, is not guaranteed or endorsed by the publisher.

## References

[1] Crow MK Olferiev M Kirou KA. Targeting of type I interferon in systemic autoimmune diseases. Transl Res. (2015) 165:296–305. 10.1016/j.trsl.2014.10.005 25468480 PMC4306610

[2] Pestka S Krause CD Walter MR. Interferons, interferon-like cytokines, and their receptors. Immunol Rev. (2004) 202:8–32. 10.1111/j.0105-2896.2004.00204.x 15546383

[3] Isaacs A Lindenmann J. Virus interference. I the interferon. Proc R Soc Lond B Biol Sci. (1957) 147:258–67. 10.1098/rspb.1957.0048 26297790

[4] Crow MK Olferiev M Kirou KA. Type I interferons in autoimmune disease. Annu Rev Pathol Mech Dis. (2019) 14:369–93. 10.1146/annurev-pathol-020117-043952 30332560

[5] Shows TB Sakaguchi AY Naylor SL Goedell DV Lawn RM. Clustering of leukocyte and fibroblast interferon genes of human chromosome 9. Science. (1982) 218:373–4. 10.1126/science.6181564 6181564

[6] Arpaia N Barton GM. Toll-like receptors: key players in antiviral immunity. Curr Opin Virol. (2011) 1:447–54. 10.1016/j.coviro.2011.10.006 22440908 PMC3311989

[7] Platanias LC. Mechanisms of type-I- and type-II-interferon-mediated signalling. Nat Rev Immunol. (2005) 5:375–86. 10.1038/nri1604 15864272

[8] Muskardin TLW Niewold TB. Type I interferon in rheumatic diseases. Nat Rev Rheumatol. (2018) 14:214–28. 10.1038/nrrheum.2018.31 29559718 PMC6625751

[9] Blasius AL Beutler B. Intracellular toll-like receptors. Immunity. (2010) 32:305–15. 10.1016/j.immuni.2010.03.012 20346772

[10] Sun L Wu J Du F Chen X Chen ZJ. Cyclic GMP-AMP Synthase is a cytosolic DNA sensor that activates the type-I interferon pathway. Science. (2013) 339:1232458. 10.1126/science.1232458 23258413 PMC3863629

[11] Schneider WM Chevillotte MD Rice CM. Interferon-stimulated genes: a complex web of host defenses. Annu Rev Immunol. (2014) 32:513–45. 10.1146/annurev-immunol-032713-120231 24555472 PMC4313732

[12] Hofer MJ Campbell IL. Type I interferon in neurological disease—The devil from within. Cytokine Growth Factor Rev. (2013) 24:257–67. 10.1016/j.cytogfr.2013.03.006 23548179

[13] Klein RS Hunter CA. Protective and pathological immunity during central nervous system infections. Immunity. (2017) 46:891–909. 10.1016/j.immuni.2017.06.012 28636958 PMC5662000

[14] Tian L Rauvala H Gahmberg CG. Neuronal regulation of immune responses in the central nervous system. Trends Immunol. (2009) 30:91–9. 10.1016/j.it.2008.11.002 19144568

[15] Daniels BP Jujjavarapu H Durrant DM Williams JL Green RR White JP. Regional astrocyte IFN signaling restricts pathogenesis during neurotropic viral infection. J Clin Invest. (2017) 127:843–56. 10.1172/JCI88720 28134626 PMC5330728

[16] Daniels BP Holman DW Cruz-Orengo L Jujjavarapu H Durrant DM Klein RS. Viral pathogen-associated molecular patterns regulate blood-brain barrier integrity via competing innate cytokine signals. MBio. (2014) 5:e01476–01414. 10.1128/mBio.01476-14 25161189 PMC4173776

[17] Müller U Steinhoff U Reis LFL Hemmi S Pavlovic J Zinkernagel RM. Functional role of type I and type II interferons in antiviral defense. Science. (1994) 264:1918–21. 10.1126/science.8009221 8009221

[18] Owens T Khorooshi R Wlodarczyk A Asgari N. Interferons in the central nervous system: a few instruments play many tunes. Glia. (2014) 62:339–55. 10.1002/glia.22608 24588027

[19] Kundu N Kumar A Corona C Chen Y Seth S Karuppagounder SS. A STING agonist preconditions against ischaemic stroke via an adaptive antiviral Type 1 interferon response. Brain Commun. (2022) 4:fcac133. 10.1093/braincomms/fcac133 35694149 PMC9175192

[20] Hosseini S Michaelsen-Preusse K Grigoryan G Chhatbar C Kalinke U Korte M. Type I interferon receptor signaling in astrocytes regulates hippocampal synaptic plasticity and cognitive function of the healthy CNS. Cell Rep. (2020) 31:107666. 10.1016/j.celrep.2020.107666 32433975

[21] Ejlerskov P Hultberg JG Wang J Carlsson R Ambjørn M Kuss M. Lack of neuronal IFN-β-IFNAR causes lewy body- and parkinson's disease-like dementia. Cell. (2015) 163:324–39. 10.1016/j.cell.2015.08.069 26451483 PMC4601085

[22] Miner JJ Daniels BP Shrestha B Proenca-Modena JL Lew ED Lazear HM. The TAM receptor Mertk protects against neuroinvasive viral infection by maintaining blood-brain barrier integrity. Nat Med. (2015) 21:1464–72. 10.1038/nm.3974 26523970 PMC4674389

[23] Mastorakos P Russo MV Zhou T Johnson K McGavern DB. Antimicrobial immunity impedes CNS vascular repair following brain injury. Nat Immunol. (2021) 22:1280–93. 10.1038/s41590-021-01012-1 34556874 PMC8488012

[24] Deczkowska A Matcovitch-Natan O Tsitsou-Kampeli A Ben-Hamo S Dvir-Szternfeld R Spinrad A. Mef2C restrains microglial inflammatory response and is lost in brain ageing in an IFN-I-dependent manner. Nat Commun. (2017) 8:717. 10.1038/s41467-017-00769-0 28959042 PMC5620041

[25] Monogue B Chen Y Sparks H Behbehani R Chai A Rajic AJ. Alpha-synuclein supports type 1 interferon signalling in neurons and brain tissue. Brain. (2022) 21:awac192. 10.1093/brain/awac192 35858675 PMC10233298

[26] Ito T Kanzler H Duramad O Cao W Liu YJ. Specialization, kinetics, and repertoire of type 1 interferon responses by human plasmacytoid predendritic cells. Blood. (2006) 107:2423–31. 10.1182/blood-2005-07-2709 16293610

[27] Serafini B Columba-Cabezas S Di Rosa F Aloisi F. Intracerebral recruitment and maturation of dendritic cells in the onset and progression of experimental autoimmune encephalomyelitis. Am J Pathol. (2000) 157:1991–2002. 10.1016/S0002-9440(10)64838-9 11106572 PMC1885753

[28] Peschke K Achleitner M Frenzel K Gerbaulet A Ada SR Zeller N. Loss of Trex1 in dendritic cells is sufficient to trigger systemic autoimmunity. J Immunol Baltim Md 1950. (2016) 197:2157–66. 10.4049/jimmunol.1600722 27511730

[29] Reinert LS Lopušná K Winther H Sun C Thomsen MK Nandakumar R. Sensing of HSV-1 by the cGAS-STING pathway in microglia orchestrates antiviral defence in the CNS. Nat Commun. (2016) 7:13348. 10.1038/ncomms13348 27830700 PMC5109551

[30] Blank T Prinz M. Type I interferon pathway in CNS homeostasis and neurological disorders. Glia. (2017) 65:1397–406. 10.1002/glia.23154 28519900

[31] Delhaye S Paul S Blakqori G Minet M Weber F Staeheli P. Neurons produce type I interferon during viral encephalitis. Proc Natl Acad Sci U S A. (2006) 103:7835–40. 10.1073/pnas.0602460103 16682623 PMC1458506

[32] Liu Y Carlsson R Ambjørn M Hasan M Badn W Darabi A. PD-L1 expression by neurons nearby tumors indicates better prognosis in glioblastoma patients. J Neurosci. (2013) 33:14231–45. 10.1523/JNEUROSCI.5812-12.2013 23986257 PMC6618508

[33] Vitner EB Farfel-Becker T Ferreira NS Leshkowitz D Sharma P Lang KS. Induction of the type I interferon response in neurological forms of Gaucher disease. J Neuroinflammation. (2016) 13:104. 10.1186/s12974-016-0570-2 27175482 PMC4866012

[34] Hammond TR Dufort C Dissing-Olesen L Giera S Young A Wysoker A. Single-Cell RNA sequencing of microglia throughout the mouse lifespan and in the injured brain reveals complex cell-state changes. Immunity. (2019) 50:253–71.e6. 10.1016/j.immuni.2018.11.004 30471926 PMC6655561

[35] Kocur M Schneider R Pulm AK Bauer J Kropp S Gliem M. IFNβ secreted by microglia mediates clearance of myelin debris in CNS autoimmunity. Acta Neuropathol Commun. (2015) 3:20. 10.1186/s40478-015-0192-4 25853624 PMC4383054

[36] Khorooshi R Mørch MT Holm TH Berg CT Dieu RT Dræby D. Induction of endogenous Type I interferon within the central nervous system plays a protective role in experimental autoimmune encephalomyelitis. Acta Neuropathol (Berl). (2015) 130:107–18. 10.1007/s00401-015-1418-z 25869642 PMC4469095

[37] Siracusa R Fusco R Cuzzocrea S. Astrocytes: role and functions in brain pathologies. Front Pharmacol. 2019 10:1114. 10.3389/fphar.2019.01114 31611796 PMC6777416

[38] Carpentier PA Begolka WS Olson JK Elhofy A Karpus WJ Miller SD. Differential activation of astrocytes by innate and adaptive immune stimuli. Glia. (2005) 49:360–74. 10.1002/glia.20117 15538753

[39] Tedeschi B Barrett JN Keane RW. Astrocytes produce interferon that enhances the expression of H-2 antigens on a subpopulation of brain cells. J Cell Biol. (1986) 102:2244–53. 10.1083/jcb.102.6.2244 2423537 PMC2114253

[40] Pfefferkorn C Kallfass C Lienenklaus S Spanier J Kalinke U Rieder M. Abortively infected astrocytes appear to represent the main source of interferon beta in the virus-infected brain. J Virol. (2016) 90:2031–8. 10.1128/JVI.02979-15 26656686 PMC4733997

[41] Kallfass C Ackerman A Lienenklaus S Weiss S Heimrich B Staeheli P. Visualizing production of beta interferon by astrocytes and microglia in brain of la crosse virus-infected mice. J Virol. (2012) 86:11223–30. 10.1128/JVI.01093-12 22875966 PMC3457137

[42] Bradl M Lassmann H. Oligodendrocytes: biology and pathology. Acta Neuropathol (Berl). (2010) 119:37–53. 10.1007/s00401-009-0601-5 19847447 PMC2799635

[43] Kapil P Butchi NB Stohlman SA Bergmann CC. Oligodendroglia are limited in type I interferon induction and responsiveness in vivo. Glia. (2012) 60:1555–66. 10.1002/glia.22375 22736486 PMC3422432

[44] Li J Hu S Zhou L Ye L Wang X Ho J. Interferon lambda inhibits herpes simplex virus type I infection of human astrocytes and neurons. Glia. (2011) 59:58–67. 10.1002/glia.21076 20878770 PMC3082435

[45] Préhaud C Mégret F Lafage M Lafon M. Virus infection switches TLR-3-positive human neurons to become strong producers of beta interferon. J Virol. (2005) 79:12893–904. 10.1128/JVI.79.20.12893-12904.2005 16188991 PMC1235836

[46] Ward LA Massa PT. Neuron-specific regulation of major histocompatibility complex class I, interferon-beta, and anti-viral state genes. J Neuroimmunol. (1995) 58:145–55. 10.1016/0165-5728(95)00005-M 7759604

[47] Chopy D Detje CN Lafage M Kalinke U Lafon M. The type I interferon response bridles rabies virus infection and reduces pathogenicity. J Neurovirol. (2011) 17:353–67. 10.1007/s13365-011-0041-6 21805057

[48] Axtell RC Raman C. Janus-like effects of type I interferon in autoimmune diseases. Immunol Rev. (2012) 248:23–35. 10.1111/j.1600-065X.2012.01131.x 22725952 PMC3383665

[49] Axtell RC de Jong BA Boniface K van der Voort LF Bhat R De Sarno P. T helper type 1 and 17 cells determine efficacy of IFN-β in multiple sclerosis and experimental encephalomyelitis. Nat Med. (2010) 16:406–12. 10.1038/nm.2110 20348925 PMC3042885

[50] Greenfield AL Hauser SL. B-cell Therapy for multiple sclerosis: entering an era. Ann Neurol. (2018) 83:13–26. 10.1002/ana.25119 29244240 PMC5876115

[51] Hemmer B Kerschensteiner M Korn T. Role of the innate and adaptive immune responses in the course of multiple sclerosis. Lancet Neurol. (2015) 14:406–19. 10.1016/S1474-4422(14)70305-9 25792099

[52] Absinta M Lassmann H Trapp BD. Mechanisms underlying progression in multiple sclerosis. Curr Opin Neurol. (2020) 33:277–85. 10.1097/WCO.0000000000000818 32324705 PMC7337978

[53] Haji Abdolvahab M Mofrad MRK Schellekens H. Interferon beta: from molecular level to therapeutic effects. Int Rev Cell Mol Biol. (2016) 326:343–72. 10.1016/bs.ircmb.2016.06.001 27572132

[54] Filipi M Jack S. Interferons in the treatment of multiple sclerosis. Int J MS Care. (2020) 22:165–72. 10.7224/1537-2073.2018-063 32863784 PMC7446632

[55] Compston A Coles A. Multiple sclerosis. Lancet Lond Engl. (2008) 372:1502–17. 10.1016/S0140-6736(08)61620-7 18970977

[56] Panitch HS Hirsch RL Haley AS Johnson KP. Exacerbations of multiple sclerosis in patients treated with gamma interferon. Lancet Lond Engl. (1987) 1:893–5. 10.1016/S0140-6736(87)92863-7 2882294

[57] Constantinescu CS Farooqi N O'Brien K Gran B. Experimental autoimmune encephalomyelitis (EAE) as a model for multiple sclerosis (MS). Br J Pharmacol. (2011) 164:1079–106. 10.1111/j.1476-5381.2011.01302.x 21371012 PMC3229753

[58] Prinz M Schmidt H Mildner A Knobeloch KP Hanisch UK Raasch J. Distinct and nonredundant in vivo functions of IFNAR on myeloid cells limit autoimmunity in the central nervous system. Immunity. (2008) 28:675–86. 10.1016/j.immuni.2008.03.011 18424188

[59] Teige I Treschow A Teige A Mattsson R Navikas V Leanderson T. IFN-β gene deletion leads to augmented and chronic demyelinating experimental autoimmune encephalomyelitis. J Immunol. (2003) 170:4776–84. 10.4049/jimmunol.170.9.4776 12707359

[60] Liu Y Hao W Letiembre M Walter S Kulanga M Neumann H. Suppression of microglial inflammatory activity by myelin phagocytosis: role of p47-PHOX-mediated generation of reactive oxygen species. J Neurosci. (2006) 26:12904–13. 10.1523/JNEUROSCI.2531-06.2006 17167081 PMC6674962

[61] Biernacki K Antel JP Blain M Narayanan S Arnold DL Prat A. Interferon beta promotes nerve growth factor secretion early in the course of multiple sclerosis. Arch Neurol. (2005) 62:563–8. 10.1001/archneur.62.4.563 15824253

[62] Caggiula M Batocchi AP Frisullo G Angelucci F Patanella AK Sancricca C. Neurotrophic factors in relapsing remitting and secondary progressive multiple sclerosis patients during interferon beta therapy. Clin Immunol Orlando Fla. (2006) 118:77–82. 10.1016/j.clim.2005.09.005 16275091

[63] Hedegaard CJ Sellebjerg F Krakauer M Hesse D Bendtzen K Nielsen CH. Interferon-beta increases systemic BAFF levels in multiple sclerosis without increasing autoantibody production. Mult Scler J. (2011) 17:567–77. 10.1177/1352458510393771 21177756

[64] Pette M Pette DF Muraro PA Farnon E Martin R McFarland HF. Interferon-beta interferes with the proliferation but not with the cytokine secretion of myelin basic protein-specific, T-helper type 1 lymphocytes. Neurology. (1997) 49:385–92. 10.1212/WNL.49.2.385 9270566

[65] Vandenbark AA Huan J Agotsch M La Tocha D Goelz S Offner H. Interferon-beta-1a treatment increases CD56bright natural killer cells and CD4+CD25+ Foxp3 expression in subjects with multiple sclerosis. J Neuroimmunol. (2009) 215:125–8. 10.1016/j.jneuroim.2009.08.007 19758707

[66] Martín-Saavedra FM González-García C Bravo B Ballester S. Beta interferon restricts the inflammatory potential of CD4+ cells through the boost of the Th2 phenotype, the inhibition of Th17 response and the prevalence of naturally occurring T regulatory cells. Mol Immunol. (2008) 45:4008–19. 10.1016/j.molimm.2008.06.006 18639934

[67] Liu Y Carlsson R Comabella M Wang J Kosicki M Carrion B. FoxA1 directs the lineage and immunosuppressive properties of a novel regulatory T cell population in EAE and MS. Nat Med. (2014) 20:272–82. 10.1038/nm.3485 24531377

[68] Calabresi PA Pelfrey CM Tranquill LR Maloni H McFarland HF. VLA-4 expression on peripheral blood lymphocytes is downregulated after treatment of multiple sclerosis with interferon beta. Neurology. (1997) 49:1111–6. 10.1212/WNL.49.4.1111 9339698

[69] Nelissen I Ronsse I Van Damme J Opdenakker G. Regulation of gelatinase B in human monocytic and endothelial cells by PECAM-1 ligation and its modulation by interferon-beta. J Leukoc Biol. (2002) 71:89–98. 10.1189/jlb.71.1.89 11781384

[70] Comabella M Río J Espejo C Ruiz de. Villa M, Al-Zayat H, Nos C, et al. Changes in matrix metalloproteinases and their inhibitors during interferon-beta treatment in multiple sclerosis. Clin Immunol Orlando Fla. (2009) 130:145–50. 10.1016/j.clim.2008.09.010 18945642

[71] Karabudak R Kurne A Guc D Sengelen M Canpinar H Kansu E. Effect of interferon β-1a on serummatrix metalloproteinase-−9 (MMP-9) and tissue inhibitor ofmatrix metalloproteinase (TIMP-1) in relapsing remittingmultiple sclerosis patients. J Neurol. (2004) 251:279–83. 10.1007/s00415-004-0285-7 15015006

[72] Boutros T Croze E Yong VW. Interferon-beta is a potent promoter of nerve growth factor production by astrocytes. J Neurochem. (1997) 69:939–46. 10.1046/j.1471-4159.1997.69030939.x 9282915

[73] Arscott WT Soltys J Knight J Mao-Draayer Y. Interferon β-1b directly modulates human neural stem/progenitor cell fate. Brain Res. (2011) 1413:1–8. 10.1016/j.brainres.2011.07.037 21855056

[74] Kappos L Hartung HP Freedman MS Boyko A Radü EW Mikol DD. Atacicept in multiple sclerosis (ATAMS): a randomised, placebo-controlled, double-blind, phase 2 trial. Lancet Neurol. (2014) 13:353–63. 10.1016/S1474-4422(14)70028-6 24613349

[75] Steri M Orrù V Idda ML Pitzalis M Pala M Zara I. Overexpression of the cytokine BAFF and autoimmunity risk. N Engl J Med. (2017) 376:1615–26. 10.1056/NEJMoa1610528 28445677 PMC5605835

[76] Gniadek P Aktas O Wandinger KP Bellmann-Strobl J Wengert O Weber A. Systemic IFN-beta treatment induces apoptosis of peripheral immune cells in MS patients. J Neuroimmunol. (2003) 137:187–96. 10.1016/S0165-5728(03)00074-2 12667663

[77] Isaksson M Ardesjö B Rönnblom L Kämpe O Lassmann H Eloranta ML. Plasmacytoid DC promote priming of autoimmune Th17 cells and EAE. Eur J Immunol. (2009) 39:2925–35. 10.1002/eji.200839179 19637225

[78] Goldmann T Zeller N Raasch J Kierdorf K Frenzel K Ketscher L. USP18 lack in microglia causes destructive interferonopathy of the mouse brain. EMBO J. (2015) 34:1612–29. 10.15252/embj.201490791 25896511 PMC4475397

[79] Gough DJ Messina NL Hii L Gould JA Sabapathy K Robertson APS. Functional crosstalk between type i and ii interferon through the regulated expression of STAT1. PLoS Biol. (2010) 8:e1000361. 10.1371/journal.pbio.1000361 20436908 PMC2860501

[80] Naves R Singh SP Cashman KS Rowse AL Axtell RC Steinman L. The Interdependent, overlapping, and differential roles of type i and ii ifns in the pathogenesis of experimental autoimmune encephalomyelitis. J Immunol. (2013) 191:2967–77. 10.4049/jimmunol.1300419 23960239 PMC3779698

[81] Karathanasis DK Rapti A Nezos A Skarlis C Kilidireas C Mavragani CP. Differentiating central nervous system demyelinating disorders: the role of clinical, laboratory, imaging characteristics and peripheral blood type I interferon activity. Front Pharmacol:. (2839). 36034800 10.3389/fphar.2022.898049 PMC9412761

[82] Tsokos GC Lo MS Costa Reis P Sullivan KE. New insights into the immunopathogenesis of systemic lupus erythematosus. Nat Rev Rheumatol. (2016) 12:716–30. 10.1038/nrrheum.2016.186 27872476

[83] Popescu A Kao AH. Neuropsychiatric systemic lupus erythematosus. Curr Neuropharmacol. (2011) 9:449–57. 10.2174/157015911796557984 22379459 PMC3151599

[84] Sarwar S Mohamed AS Rogers S Sarmast ST Kataria S Mohamed KH. Neuropsychiatric systemic lupus erythematosus: a 2021 update on diagnosis, management, and current challenges. Cureus 13(9):e. (17969). 10.7759/cureus.17969 34667659 PMC8516357

[85] Wen J Stock AD Chalmers SA Putterman C. The role of B cells and autoantibodies in neuropsychiatric lupus. Autoimmun Rev. (2016) 15:890–5. 10.1016/j.autrev.2016.07.009 27389531 PMC4982790

[86] Fredi M Bianchi M Andreoli L Greco G Olivieri I Orcesi S. Typing TREX1 gene in patients with systemic lupus erythematosus. Reumatismo. (2015) 67:1–7. 10.4081/reumatismo.2015.782 26150267

[87] Bennett L Palucka AK Arce E Cantrell V Borvak J Banchereau J. Interferon and granulopoiesis signatures in systemic lupus erythematosus blood. J Exp Med. (2003) 197:711–23. 10.1084/jem.20021553 12642603 PMC2193846

[88] Tanaka Y Tummala R. Anifrolumab, a monoclonal antibody to the type I interferon receptor subunit 1, for the treatment of systemic lupus erythematosus: an overview from clinical trials. Mod Rheumatol. (2021) 31:1–12. 10.1080/14397595.2020.1812201 32814461

[89] Kirou KA Dall‘Era M Aranow C Anders HJ. Belimumab or anifrolumab for systemic lupus erythematosus? A risk-benefit assessment. Front Immunol. (2022) 13:980079. 10.3389/fimmu.2022.980079 36119023 PMC9472122

[90] Shiozawa S Kuroki Y Kim M Hirohata S Ogino T. Interferon-alpha in lupus psychosis. Arthritis Rheum. (1992) 35:417–22. 10.1002/art.1780350410 1373622

[91] Santer DM Yoshio T Minota S Möller T Elkon KB. Potent induction of IFN-alpha and chemokines by autoantibodies in the cerebrospinal fluid of patients with neuropsychiatric lupus. J Immunol Baltim Md 1950. (2009) 182:1192–201. 10.4049/jimmunol.182.2.1192 19124763 PMC2745922

[92] Lindblom J Mohan C Parodis I. Biomarkers in neuropsychiatric systemic lupus erythematosus: a systematic literature review of the last decade. Brain Sci. (2022) 12:192. 10.3390/brainsci12020192 35203955 PMC8869794

[93] Karnopp TE Chapacais GF Freitas EC Monticielo OA. Lupus animal models and neuropsychiatric implications. Clin Rheumatol. (2021) 40:2535–45. 10.1007/s10067-020-05493-7 33155159

[94] Perry D Sang A Yin Y Zheng YY Morel L. Murine models of systemic lupus erythematosus. J Biomed Biotechnol. (2011) 2011:e271694. 10.1155/2011/271694 21403825 PMC3042628

[95] Nocito C Lubinsky C Hand M Khan S Patel T Seliga A. Centrally acting angiotensin-converting enzyme inhibitor suppresses type i interferon responses and decreases inflammation in the periphery and the CNS in lupus-prone mice. Front Immunol. (2020) 11:573677. 10.3389/fimmu.2020.573677 33042154 PMC7522287

[96] Aicardi J Goutières F A. Progressive familial encephalopathy in infancy with calcifications of the basal ganglia and chronic cerebrospinal fluid lymphocytosis. Ann Neurol. (1984) 15:49–54. 10.1002/ana.410150109 6712192

[97] Campbell IL Krucker T Steffensen S Akwa Y Powell HC Lane T. Structural and functional neuropathology in transgenic mice with CNS expression of IFN-alpha. Brain Res. (1999) 835:46–61. 10.1016/S0006-8993(99)01328-1 10448195

[98] Crow YJ Stetson DB. The type I interferonopathies: 10 years on. Nat Rev Immunol. (2022) 22, 471–83. 10.1038/s41577-021-00633-9 34671122 PMC8527296

[99] Volpi S Picco P Caorsi R Candotti F Gattorno M. Type I interferonopathies in pediatric rheumatology. Pediatr Rheumatol Online J. (2016) 14:35. 10.1186/s12969-016-0094-4 27260006 PMC4893274

[100] Rice GI Melki I Frémond ML Briggs TA Rodero MP Kitabayashi N. Assessment of type I interferon signaling in pediatric inflammatory disease. J Clin Immunol. (2017) 37:123–32. 10.1007/s10875-016-0359-1 27943079 PMC5325846

[101] d'Angelo DM Di Filippo P Breda L Chiarelli F. Type I Interferonopathies in Children: An Overview. Front Pediatr. (2021) 9:631329. 10.3389/fped.2021.631329 33869112 PMC8044321

[102] McDonough A Lee RV Weinstein JR. Microglial interferon signaling and white matter. Neurochem Res. (2017) 42:2625–38. 10.1007/s11064-017-2307-8 28540600 PMC5777301

[103] Gonçalves FG Caschera L Teixeira SR Viaene AN Pinelli L Mankad K. Intracranial calcifications in childhood: Part 1. Pediatr Radiol. (2020) 50:1424–47. 10.1007/s00247-020-04721-1 32734340

[104] Crow YJ Rehwinkel J. Aicardi-Goutieres syndrome and related phenotypes: linking nucleic acid metabolism with autoimmunity. Hum Mol Genet. (2009) 18:R130–136. 10.1093/hmg/ddp293 19808788 PMC2758706

[105] Zhang X Bogunovic D Payelle-Brogard B Francois-Newton V Speer SD Yuan C. Human intracellular ISG15 prevents interferon-α/β over-amplification and auto-inflammation. Nature. (2015) 517:89–93. 10.1038/nature13801 25307056 PMC4303590

[106] Rodero MP Tesser A Bartok E Rice GI Della Mina E Depp M. Type I interferon-mediated autoinflammation due to DNase II deficiency. Nat Commun. (2017) 8:2176. 10.1038/s41467-017-01932-3 29259162 PMC5736616

[107] Schorr S Legum C Ochshorn M. Spondyloenchondrodysplasia. Enchondromatomosis with severe platyspondyly in two brothers. Radiology. (1976) 118:133–9. 10.1148/118.1.133 1244645

[108] Liu Y Ramot Y Torrelo A Paller AS Si N Babay S. Mutations in proteasome subunit β type 8 cause chronic atypical neutrophilic dermatosis with lipodystrophy and elevated temperature with evidence of genetic and phenotypic heterogeneity. Arthritis Rheum. (2012) 64:895–907. 10.1002/art.33368 21953331 PMC3278554

[109] Crow YJ Manel N. Aicardi-Goutières syndrome and the type I interferonopathies. Nat Rev Immunol. (2015) 15:429–40. 10.1038/nri3850 26052098

[110] Rice GI Forte GMA Szynkiewicz M Chase DS Aeby A Abdel-Hamid MS. Assessment of interferon-related biomarkers in Aicardi-Goutières syndrome associated with mutations in TREX1, RNASEH2A, RNASEH2B, RNASEH2C, SAMHD1, and ADAR: a case-control study. Lancet Neurol. (2013) 12:1159–69. 24183309 10.1016/S1474-4422(13)70258-8 PMC4349523

[111] Freitas EC de Oliveira MS Monticielo OA. Pristane-induced lupus: considerations on this experimental model. Clin Rheumatol. (2017) 36:2403–14. 10.1007/s10067-017-3811-6 28879482

[112] Stetson DB Ko JS Heidmann T Medzhitov R. Trex1 Prevents cell-intrinsic initiation of autoimmunity. Cell. (2008) 134:587–98. 10.1016/j.cell.2008.06.032 18724932 PMC2626626

[113] van Heteren JT Rozenberg F Aronica E Troost D Lebon P Kuijpers TW. Astrocytes produce interferon-alpha and CXCL10, but not IL-6 or CXCL8, in Aicardi-Goutières syndrome. Glia. (2008) 56:568–78. 10.1002/glia.20639 18240301

[114] Cuadrado E Jansen MH Anink J De Filippis L Vescovi AL Watts C. Chronic exposure of astrocytes to interferon-α reveals molecular changes related to Aicardi–Goutières syndrome. Brain. (2013) 136:245–58. 10.1093/brain/aws321 23365100

[115] Crow YJ Vanderver A Orcesi S Kuijpers TW Rice GI. Therapies in aicardi–goutières syndrome. Clin Exp Immunol. (2014) 175:1–8. 10.1111/cei.12115 23607857 PMC3898548

[116] Ramantani G Maillard LG Bast T Husain RA Niggemann P Kohlhase J. Epilepsy in aicardi–goutières syndrome. Eur J Paediatr Neurol. (2014) 18:30–7. 10.1016/j.ejpn.2013.07.005 24011626

[117] Lindahl H Bryceson YT. Neuroinflammation associated with inborn errors of immunity. Front Immunol. (2022) 12:827815. 10.3389/fimmu.2021.827815 35126383 PMC8807658

[118] Martin-Fernandez M García-Morato MB Gruber C Loza SM Malik MNH Alsohime F. Systemic type I IFN inflammation in human ISG15 deficiency leads to necrotizing skin lesions. Cell Rep. (2020) 31:107633. 10.1016/j.celrep.2020.107633 32402279 PMC7331931

[119] Pawaria S Moody K Busto P Nündel K Choi CH Ghayur T. Cutting edge: DNase II deficiency prevents activation of autoreactive B cells by double-stranded DNA endogenous ligands. J Immunol. (2015) 29 194:1403–7. 10.4049/jimmunol.1402893 25601924 PMC4323726

[120] Utsumi T Okada S Izawa K Honda Y Nishimura G Nishikomori R. A case with spondyloenchondrodysplasia treated with growth hormone. Front Endocrinol. (2017) 8:157. 10.3389/fendo.2017.00157 28740483 PMC5502255

[121] Patel PN Hunt R Pettigrew ZJ Shirley JB Vogel TP de Guzman MM. Successful treatment of chronic atypical neutrophilic dermatosis with lipodystrophy and elevated temperature (CANDLE) syndrome with tofacitinib. Pediatr Dermatol. (2021) 38:528–9. 10.1111/pde.14517 33512037

[122] Goetzke CC Ebstein F Kallinich T. Role of proteasomes in inflammation. J Clin Med. (2021) 10:1783. 10.3390/jcm10081783 33923887 PMC8072576

